# Th9 Cell Differentiation and Its Dual Effects in Tumor Development

**DOI:** 10.3389/fimmu.2020.01026

**Published:** 2020-05-20

**Authors:** Tao Chen, Jufeng Guo, Zhenhai Cai, Binghao Li, Lingling Sun, Yingying Shen, Shengdong Wang, Zhan Wang, Zenan Wang, Yucheng Wang, Hao Zhou, Zhijian Cai, Zhaoming Ye

**Affiliations:** ^1^Department of Orthopedics, Musculoskeletal Tumor Center, The Second Affiliated Hospital of Zhejiang University School of Medicine, Hangzhou, China; ^2^Institute of Orthopedic Research, Zhejiang University, Hangzhou, China; ^3^Department of Breast Surgery, Affiliated Hangzhou First People's Hospital, Zhejiang University School of Medicine, Hangzhou, China; ^4^Department of Orthopedics Surgery, The Second Affiliated Hospital of Jiaxing University, Jiaxing, China; ^5^Institute of Immunology, Zhejiang University School of Medicine, Hangzhou, China

**Keywords:** Th9 cells, IL-9, cancer, cancer immunotherapy, adoptive cell therapy, regulation of CD4 helper T cell differentiation

## Abstract

With the improved understanding of the molecular pathogenesis and characteristics of cancers, the critical role of the immune system in preventing tumor development has been widely accepted. The understanding of the relationship between the immune system and cancer progression is constantly evolving, from the cancer immunosurveillance hypothesis to immunoediting theory and the delicate balance in the tumor microenvironment. Currently, immunotherapy is regarded as a promising strategy against cancers. Although adoptive cell therapy (ACT) has shown some exciting results regarding the rejection of tumors, the effect is not always satisfactory. Cellular therapy with CD4^+^ T cells remains to be further explored since the current ACT is mainly focused on CD8^+^ cytotoxic T lymphocytes (CTLs). Recently, Th9 cells, a subgroup of CD4^+^ T helper cells characterized by the secretion of IL-9 and IL-10, have been reported to be effective in the elimination of solid tumors and to exhibit superior antitumor properties to Th1 and Th17 cells. In this review, we summarize the most recent advances in the understanding of Th9 cell differentiation and the dual role, both anti-tumor and pro-tumor effects, of Th9 cells in tumor progression.

## Introduction

In recent years, immunotherapy has become a promising strategy for the treatment of solid tumors and hematologic malignancies. Adoptive cell therapy (ACT) is an important branch of immunotherapy, which has been proven to be successful in inducing an objective clinical response in some tumors, such as melanoma, ovarian cancer, and colorectal cancer (1–3). However, the current ACT is mainly focused on CD8^+^ cytotoxic T lymphocytes (4), while the anti-tumor efficacy of CD4^+^ T cells has not been fully explored. CD4^+^ helper T cells are established key components of the adaptive immunity and shape anti-cancer immunity in different models. CD4^+^ helper T cells are also known for their high plasticity and the ability to differentiate into different subsets with various functions. The designation of the subpopulations, including but not limited to Th1, Th2, Th17, and Treg cells, is determined by the expression pattern of specific cytokines and transcription factors.

In 1986, Mosmann and Coffman defined two subsets of CD4^+^ T cells, Th1 and Th2 cells, for the first time (5). Th1 cells are generally considered to protect the host against tumor development by secreting multiple cytokines, including IFN-γ and IL-2, and enhancing the recruitment and activity of CD8^+^ T cells and NK T cells (6–8). Thus, Th1 cells play a vital role in shaping the anti-tumor immune response (9). However, tumor-specific Th1 cells induced *in vitro* was found to exhibit a more exhausted phenotype, and a lack of persistence *in vivo* (10). The evidences regarding the role of Th2 cells in anti-tumor activities are conflicting. Th2 cells are known to eliminate tumor cells by recruiting tumoricidal eosinophils and macrophages to the tumor microenvironment due to the secretion of IL-4 and IL-13 cytokines (11, 12). However, it has been reported that Th2 cells secrete cytokines that contribute to the suppression of anti-tumor immune system (13, 14). Matsuda and Sharma observed that Th2 cells-derived IL-10 decreased the MHC-I expression and mediated the inhibition of DC activity, mainly antigen processing and presentation, leading to tumor progression (15–17). In addition, IL-10 may activate regulatory T cells, which are characterized by highly immunosuppressive properties (18). This effect has been supported by several studies, which demonstrated that the neutralization of IL-10 successfully restored or boosted the anti-tumor immune response (19). The role of Th17 cells in tumor immunity may be paradoxical depending on the tumor type. For example, it was found that IL-17 derived from Th17 cells promoted angiogenesis and correlated with a poor prognosis in colorectal carcinoma (20), while Muranski demonstrated that tumor-specific Th17 cells were superior to tumor-specific Th1 cells in the eradication of established melanoma (21). This therapeutic effect was mainly dependent on IFN-γ, while IL-17A and IL-23 only marginally contributed to this effect. Additionally, Martin-Orozco reported that Th17 cells were capable of promoting dendritic cell (DC) infiltration and antigen presentation, which finally elicited a robust CD8^+^ T cell response in a mouse melanoma model (22). Besides, Amedei et al. reported the opposing role of Tregs and Th17 cells in pancreatic cancer (PC) (23). They first discovered that the level of α-Enolase (ENO1)-specific Treg cells in PC patients increased while the level of intra-tumoral Th17 cells decreased. To better characterize the effector functions of ENO1-specific Treg and Th17 cells, they isolated these cells from PC patients and found that IL-17/IFN-γ double positive Th17 cells could efficiently kill target cells *in vitro*, while ENO1-specific Tregs inhibit effector T cells (Teff). This was consistent with their observation that patients with a low ENO1-specific Treg/Teff ratio survived longer than those with a high ratio. These results indicated that Th17 cells exerted an anti-tumor function while Tregs promoted the development of PC (23).

In 2008, Veldhoen and Dardalhon reported that TGF-β and IL-4 induced the generation of predominantly Forkhead box p3 (Foxp3)^−^ IL-9^+^ IL-10^+^ T cells, which mainly secreted IL-9 and IL-10 and were designated as a novel subset of the CD4^+^ Th cells called Th9 cells (24, 25). Initially, Th9 cells were thought to contribute to numerous autoimmune diseases, including multiple sclerosis (MS), inflammatory bowel disease (IBD), rheumatoid arthritis (RA), systemic lupus erythematosus (SLE), and psoriasis (26). However, further studies showed that Th9 cells harbored potent anti-cancer properties in solid tumors (27–30). More importantly, Lu and Yi demonstrated that Th9 cells were less exhausted than Th1 cells but were highly cytolytic and possessed a hyperproliferative phenotype similar to that of Th17 cells (10). Thus, Th9 cell is a potential candidate for ACT therapy against cancers. In this review, we summarize the studies focused on the differentiation of Th9 subsets and reveal both the positive and negative relationships between Th9 cells and tumor development.

## Generation and Differentiation of Th9 Cells

Initially, IL-9 was thought to be a Th2-specific cytokine, whereas Veldohen et al. incidentally found that Th2 cells could be reprogrammed and differentiated into a distinct Th subset that preferentially secreted IL-9 under stimulation by TGF-β and IL-4 (25). Functionally, Th cells can be distinguished based on their cytokine-producing profiles, the expression of fate-determining transcription factors, and cluster differential markers (31). Th9 cells are characterized by the secretion of IL-9 and IL-10, whereas Th2 cells produce IL-4, IL-5, IL-13, and a modest amount of IL-9. Besides, Th9 and Th2 cells are epigenetically imprinted by PU.1 [also known as Spi-1 proto-oncogene (SPI1)] and GATA binding protein 3 (GATA3), respectively (5, 24, 32, 33). What's more, human Th9 cells express CD183 (CXCR3), CD193 (CCR3), and CD196 (CCR6), but not CD194^+^(CCR4^+^) or D294 (CRTH2), which are expressed on the surface of Th2 cells (34, 35). Thus, Th9 cells are identified as a distinct Th cell lineage rather than a subgroup of Th2 cells even though both Th9 cells and Th2 cells are associated with the pathogenesis of many autoimmune diseases and protection from helminth infections (36–38).

## Transcription Factors Involved in the Differentiation of Th9 Cells and Other Th Subsets

**Figure 1:**
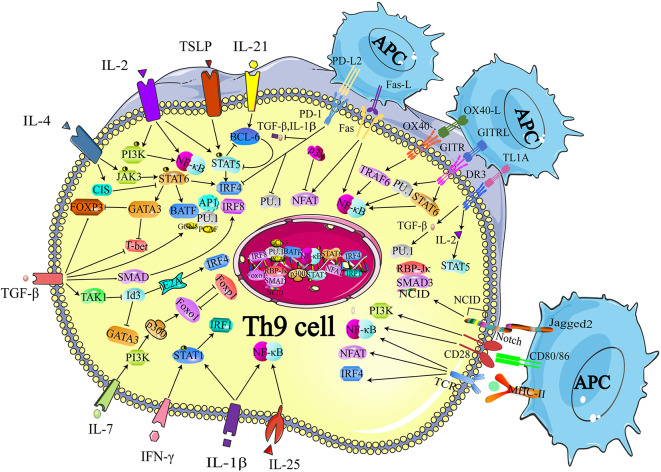
Transcriptional regulation of Th9 cell differentiation. The development of Th9 cells mainly relies on TCR-NFAT/NF-κB signals, IL-2-STAT5 signals, TGF-β-SMAD signals, and IL-4-STAT6 signals. Some other cytokines are also identified to synergistically enhance Th9 cell development, such as IL-1, IL-25, IL-7, IL-21, while IFN-γ is reported to inhibit IL-9 production through STAT-1. These signals also induce expression of the GATA3, IRF 4, IRF8, IRF1, PU.1, and BATF, which contribute to the chromatin modification at *IL-9* and *IL-21* locus. Many proteins or small molecules are reported to activate the NFAT and NF-κB, such as OX40, GITR, and TL1A. TCR, T cell receptor; NFAT, nuclear factor of activated T cells; NF-κB, nuclear factor-κB; STAT, Signal Transducer and Activator of Transcription; TGF-β, transforming growth factor-β; GATA-3, GATA-binding protein 3; IRF, transcription factors interferon (IFN)-regulatory factor; BATF, basic leucine zipper transcription factor, ATF like; NICD, Notch intracellular domain, RBP-Jk, recombination signal binding protein for immune globulin kJ region; OX40, Tumor necrosis factor receptor superfamily member 4; GITR, glucocorticoid-induced tumor necrosis factor receptor (TNFR)-related protein; OX40, Tumor necrosis factor receptor superfamily member 4. Figures were produced using Servier Medical Art https://smart.seriver.com.

Although IL-9 was considered as a cytokine of Th9 cells, other Th subsets were also reported with the production of IL-9, including Th2, Th17, and Tregs (39, 40). The regulatory network of transcription factors in Th9 cells is quite intricate since a large proportion of the transcription factors expressed in Th9 cells are also expressed in other Th subsets. Thus, in this section, we simply summarize the critical transcription factors that are involved in the differentiation of Th9 cells as well as other Th subsets, such as Signal Transducer and Activator of Transcription 6 (STAT6), PU.1, GATA3, nuclear factor of activated T cells (NFAT), interferon regulatory factor 4 (IRF-4), and drosophila mothers against decapentaplegic protein (SMAD) (Figure 1) (24, 41–43). These transcription factors have divergent roles in different cells. For example, STA6, PU.1, and GATA3 are involved in the differentiation of both Th9 and Th2 cells. STAT6 induces the expression of GATA3 after phosphorylated by IL-4 signaling. The primary function of IL-4R-STAT6-GATA-3 in Th9 cells is to counteract the TGF-β-induced Foxp3 expression, while the same axis is responsible for inducing the expression of IL-4 in Th2 cells (24, 25). PU.1, an EST family transcription factor, is highly expressed in Th9 cells compared with Th2 cells. Overexpression of PU.1 upregulates the secretion of IL-9 but constrains Th2 cell differentiation (44). NFAT1 interacts with CBP/P300 histone acetyltransferase proteins and promotes IL-9 expression (42, 43). In addition, NFAT1 induces IL-4 and IL-10 expression in Th2 cells. And in Th1 cells, NFAT1 cooperates with STAT1 and activator protein 1 (AP1), binding to the IFN-γ promoter region (45). SMAD was reported to be associated with the regulation of Tregs, Th17 cells as well as Th9 cells. TGF-β signaling phosphorylates SMAD2 and SMAD3 (46), which are redundantly essential for TGF-β-mediated induction of Foxp3-expressing regulatory T cells (47). SMAD2 and SMAD4 are necessary for the differentiation of Th9 cells (48). However, it is SMAD4, neither SMAD2 nor SMAD3, required for Th17 cell differentiation (49, 50). IRF4, another target gene of STAT6, is involved in Th9 cell differentiation, which is also required for Th1, Th2, and Th17 cell differentiation. IRF4 cooperates with B cell-activating transcription factor-like (BATF) and binds to the *IL-9* locus, promoting Th9 cell development (41, 51). While in Th2 cells, IRF4 cooperates with NFAT1 and NFAT2 to modulate IL-4 expression (52, 53). Besides, deficiency of IRF-4 was reported to be associated with defects in the up-regulation of GATA3 in Th2 cells as well as the compromised differentiation of IL-12-induced Th1 cells, indicating that IRF-4 was also required for Th1 cell differentiation (54). Additionally, the specific interaction between NFAT1 and IRF4 was detected in Th1 cells (53).

## The Role OF IL-4 Signaling in Th9 Cell Differentiation

STAT6 is a critical signaling component of IL-4-induced Th9 cell differentiation. The recruitment of STAT6 requires the IL-4Rα-induced activation of Janus kinase (JAK)1 and JAK3 (39). Dardalhon and colleagues found that STAT6-deficient and GATA3-deficient mice could no longer induce IL-9-producing cells in the presence of TGF-β plus IL-4, and more importantly, they proved that STAT6 was involved in IL-4-mediated Foxp3 inhibition induced by TGF-β. They also demonstrated that Foxp3 physically associated with GATA3 and inhibited the transactivation of Th2 genes (24). In conclusion, on the one hand, IL-4 phosphorylates STAT6, and p-STAT6 directly binds to the *IL-9* locus. On the other hand, TGF-β induces the expression of Foxp3, which can be inhibited by IL-4-induced p-STAT6, suppressing Th9 cell differentiation. Thus, IL-4 and TGF-β act in concert to regulate Th9 cell differentiation (Figure 1).

Goswami et al. confirmed that IL-4 and p-STAT6 also facilitated the transcription of *Irf4*, which promoted IL-9 production (Figure 1) (41, 55). Staudt and colleagues reported that IL-9 production and Th9 cell differentiation were hampered *in vitro* when IRF4-deficient naïve CD4^+^ T cells or wild-type naïve CD4^+^ T cells were treated with IRF4-specific siRNA. They also performed chromatin-immunoprecipitation (ChIP) analyses and revealed that IRF4 bound directly to the IL-9 promoter and increased *Il9* transcription (41). Moreover, by inducing IRF4 expression, TGF-β and especially IL-4 neutralized the inhibitory effect of endogenously produced IFN-γ, while deletion of STAT6 and IRF4 led to elevated IFN-γ and T-box expressed in T cells (T-bet) expression. Therefore, it is likely that IRF4 not only promotes Th9 cell differentiation, but also inhibits the expression of Th1 cell-associated transcription factors, which may impede the differentiation of Th9 cells.

Additionally, IRF4 cooperates with AP1 complex and forms a heterodimer with a basic leucine zipper transcription factor, BATF, which functions as a transcriptional module, increasing IL-9 secretion (Figure 1) (51). The lack of BATF impaired IL-9 production and Th9 cell differentiation, while naïve T cells from BATF-transgenic mice exhibited higher IL-9 production under Th9 cell-inducing conditions (51).

GATA3 is a target gene of STAT6 and a critical regulator of Th2 cells. However, several groups have reported that GATA3 also functioned in a STAT6-independent manner. For example, Amsen et al. and Fang et al. suggested that Notch-dependent signaling regulated the transcription of GATA3 (56, 57). Fang and colleagues proved that Notch preferentially induced the expression of the *Gata3* transcript that included exon 1a sequences and Notch directly associated with *Gata3* through CSL (gene name *Rbpj*)-binding sites (57). This result was confirmed by Amsen and colleagues. They found that Notch specifically activated the upstream GATA3 promoter in an RBP-J dependent manner, as Notch responsiveness of exon 1a was abrogated in RBP-J deficient CD4^+^ T cells (56).

IL-4 has also been reported to negatively regulate Th9 cell differentiation. Suppressor of cytokine signaling (SOCS) family proteins were responsible for repressing STAT signaling and protecting the host from the potential damage caused by the overactivation of STATs. Dong and colleagues reported that cytokine-induced SH-2 protein (CIS), a member of the SOCS family that cloud be induced by IL-4, repressed the activation of STAT proteins, including STAT3, STAT5, and STAT6 (Figure 1) (58). Consistent with this finding, these authors observed that CIS-deficient mice exhibited severe airway allergic disease compared with normal mice. Further experiments revealed that Th2 and Th9 cell differentiation were remarkably promoted in the absence of CIS in T cells, which seemed to be a reasonable explanation for the *in vivo* findings.

## The Role OF TGF-β Signaling in Th9 Cell Differentiation

The TGF-β signaling pathway is involved in the transcriptional regulation of Th9 cell differentiation (24, 25, 59). It is well-established that TGF-β induces the expression of Foxp3 and contributes to the development of Treg cells. The SMAD protein family is composed of three proteins, SMAD2, SMAD3, and SMAD4, acting as signaling intermediates of the TGF-β superfamily. It has been verified that deficiency of SMAD2 or SMAD4 in T cells resulted in the loss of IL-9 expression with the enrichment of the repressive chromatin modification of histone H3K27 trimethylation (48). Elyaman et al. found that Notch and SMAD3 were involved in Th9 cell differentiation and participated in regulating the immune response under TGF-β-based polarizing conditions (Figure 1) (60). The Notch proteins (Notch1–Notch4) are single-pass receptors that activated by the Delta-like (consisting of DLL1, DLL3, and DLL4) and Jagged/Serrate (Jagged1 and Jagged2) families of membrane-bound ligands (61). These authors revealed that it was Jagged2 ligation but not the Delta-like 1 that was responsible for IL-9 production (Figure 1). Specifically, they found that the development of Th9 cells was impaired when Notch1 and Notch2 were conditionally deleted. The Notch1 intracellular domain (NICD1) was responsible for the recruitment of SMAD3. SMAD3, together with recombining binding protein (RBP)-Jκ, bound to the IL-9 promoter and increased IL-9 production (Figure 1).

Further studies revealed that TGF-β contributed to preventing the expression of T-bet and inducing the expression of the ETS-family transcription factor PU.1, which was encoded by *Sfpi1* (Figure 1). Chang et al. showed that PU.1 deficiency impaired IL-9 production, whereas ectopic PU.1 expression promoted IL-9 production (62). PU.1 bound to the IL-9 promoter and then recruited the histone acetyltransferases (HAT) proteins Gcn5 and PCAF, increasing chromatin accessibility at the *IL-9* locus. Hence, the binding to other transcription factors was facilitated and the transcription of the *Il9* gene was initiated (63). Interestingly, the expression of *Sfpi1* was not affected when SMAD-deficient T cells were cultured under Th9-skewing conditions, which indicated that PU.1 might be regulated by SMAD-independent signaling mechanisms (64).

Moreover, Tamiya et al. revealed that SMAD2 or SMAD3 physically interacted with IRF4 to cooperatively activate the IL-9 promoter (65). These data helped to explain how the IL-4-STAT6-induced activation of IRF4 and the TGFβ-driven activation of SMADs worked together to transactivate the *Il9* gene. IRF8 is an IRF4 homolog, indicating the possible involvement of IRF8 in Th9 cell differentiation. Etienne Humblin et al. proved that IRF8 was induced via the TGF-β signaling pathway and contributed to the development of Th9 cells both *in vivo* and *in vitro* (Figure 1) (66). They found that IRF8 alone was insufficient to induce the expression of IL-9 and IL-21, and that the cooperation of IRF4, PU.1, and BATF was required. In addition, they observed that IRF8 directly interacted with ETS variant 6 (ETV6), a repressive transcription factor that functioned through epigenetic modification (67), and then inhibited IL-4 expression, which was critical in mediating the differentiation of Th2 cells (68). Their work illustrated two roles of IRF8 in the regulation of Th9 cell differentiation and the expression of Th9 cell cytokines. On the one hand, IRF8 interacted with IRF4, PU.1, and BATF, and formed a large complex to induce Th9 cell polarization and related cytokine expression, especially IL-9 and IL-21. On the other hand, IRF8 acted synergistically with ETV6 and repressed the expression of IL-4 via epigenetic modulation, thereby impeding transmission from Th9 cells to Th2 cells.

Hiroko Nakatsukasa et al. also reported the TGF-β1-induced SMAD-independent induction of Th9 cell differentiation (69). First, they found that deletion of Id3, an E-box transcription factor inhibitor, increased IL-9 production. They proved that TGF-β1 and IL-4 downregulated Id3. To explore the underlying mechanism of the TGF-β1-induced SMAD-independent induction of Th9 cell differentiation, they treated naïve CD4^+^ T cells with 5z-7-oxozeaenol, a TAK1 inhibitor, and found that Th9 cell differentiation was almost completely blocked, whereas the differentiations of Th1, Th17, and Treg cells were not affected, suggesting a specific function of TAK1 in Th9 cell differentiation (Figure 1). Further experiments showed that the suppression of Id3 was significantly reversed after exposure to a TAK1 inhibitor for 24 h. It has been reported that Id3 formed a complex with E2A and prevented E2A binding to its target genes, thus decreasing the transcription activity of certain genes (70). Additionally, it has been proved that Id3 deficiency increased GATA-3 expression (Figure 1) (71). They confirmed that E2A and GATA3 were enriched in the IL-9 promoter region in response to TGF-β together with IL-4 and the mutation of the E-box and GATA-3-binding sites impaired IL-9 promoter activity (69). In conclusion, the study of Hiroko Nakatsukasa identified the function of the TAK1–Id3–E2A–GATA-3 pathway in Th9 cell differentiation. Similar to Id3, Liu and colleagues reported that SIRT1, an NAD^+^-dependent histone deacetylase, acted as a negative regulator of Th9 cell differentiation through an mTOR-HIF1α-dependent signaling pathway (72).

Recently, Kerzerho et al. verified that programmed cell death ligand (PD-L) 2 negatively regulated Th9 cell development in chronic airway hyperreactivity (AHR). They found that the deficiency of PD-L2 in an *Aspergillus fumigatus*-induced AHR model increased Th9 cell differentiation with the upregulation of PU.1, IRF4 T, TGF-β, and IL-1α, whereas the number of IL-4-producing Th2 cells was unaffected (Figure 1) (73).

## The Role OF T Cell Receptor (TCR) and Co-Stimulation Signaling in Th9 Cell Differentiation

The TCR signaling pathway is important for the generation of Th9 cells and the secretion of IL-9. Jash revealed that the TCR-mediated activation of NFAT acted synergistically with nuclear factor kB (NF-κB) p65 to regulate Th9 cell differentiation (Figure 1, Table 1). NFAT1 increased chromatin accessibility through its interactions with CBP/P300 histone acetyltransferase proteins and then increased the recruitment of NF-κB to the IL-9 promoter, promoting IL-9 expression (Figure 1) (42). TCR signaling also increased the expression of IRF4, which played a pivotal role in the differentiation of CD4^+^ T cells, not only Th9 cells but also Th2 and Th17 cells (41, 79–81).

**Table 1:** T cell receptor and co-stimulatory molecules and soluble factors that are involved in Th9 cell differentiation.

**Receptor on T cells**	**Ligand on APC**	**Main signaling pathways**	**Effects on Th9 differentiation**	**References**
TCR	Peptide–MHC class II	NFAT and NF-κB	Promotes	(42)
CD28	CD80 or CD86	PI3K and NF-κB	Promotes	(59)
OX40	OX40L	TRAF6 and NF-κB (p52-RelB)	Promotes	(74)
GITR	GITR-L	NF-κB (p50-RelA)	Promotes	(74, 75)
Fas	Fas-L	NF-κB and NFAT1	Promotes	(76)
Notch	Jagged 2	NICD1	Promotes	(60, 61)
DR3	TL1A	IL-2-STAT5 and PU.1	Promotes	(77, 78)
PD1	PDL2	SHP2	Inhibits	(73)

*TCR, T cell receptor; NFAT, nuclear factor of activated T cells; NF-κB, nuclear factor-κB; PI3K, phosphoinositide 3-kinase; OX40, Tumor necrosis factor receptor superfamily member 4; OX40L, OX40 ligand; GITR, glucocorticoid-induced tumor necrosis factor receptor (TNFR)-related protein; GITRL, glucocorticoid-induced tumor necrosis factor receptor (TNFR)-related protein ligand; TRAF, TNF receptor-associated factor; NICD1, Notch 1 intracellular domain; TL1A, TNF-liked ligand 1A; DR3, death recetpor 3; PD1, programmed cell death protein 1; PDL2, programmed cell death 1 ligand 2; SHP2, SRC homology 2 domain-containing protein tyrosine phosphatase 2*.

Many proteins or small molecules have been reported to activate the NFAT and NF-κB (Table 1). For example, Fas, a member of the tumor necrosis factor receptor (TNF-R) family, plays a pivotal role in T cell homeostasis by inducing activation-induced cell death (AICD) (82). Recently, Shen et al. reported that non-apoptotic Fas signaling contributed to Th9 cell differentiation via the cooperation of NFAT1 and NF-κB, which was activated by Ca2^+^-dependent PKC-β activation (76). More interestingly, they identified p38 as a negative regulator of Fas-mediated Th9 cell differentiation by inhibiting the function of NFAT1 (Figure 1).

In addition, Tumor necrosis factor receptor superfamily member 4 (OX40) and glucocorticoid-induced tumor necrosis factor receptor (TNFR)-related protein (GITR) are T cell-costimulatory molecules of the TNF receptor superfamily (74, 83). Xiao et al. verified that both OX40 and GITR-derived signaling favored the differentiation of Th9 cells via different transcription factors but restrained Foxp3^+^ Tregs development (Table 1) (75, 84). Specifically, they found that OX40 recruited TRAF6, a member of the TRAF protein family that acted as an adaptor for the activation of NF-κB signaling. TRAF6 triggered the induction of NF-κB-inducing kinase (NIK) in CD4^+^ T cells and led to the activation of the non-canonical NF-κB pathway (p52-RelB) since p52 and RelB were found to specifically accumulated under OX40 treatment (Figure 1) (75). In contrast to OX40-induced Th9 cells, which were independent of PU.1, STAT5, STAT6, and STAT3, GITR-induced IL-9-producing cells required the involvement of STAT6, BATF, PU.1, and IRF-4, and the activation of the canonical NF-κB pathway (p50-RelA). These proteins bound to the Foxp3 promoter and recruited histone deacetylases to the *Foxp3* locus to remodel chromatin accessibility, inhibiting Foxp3 expression and conversely increasing the expression of IL-9 (Figure 1) (84). Kim et al. confirmed this mechanism and additionally found that GITR activation enhanced the antitumor effects of Th9 cells by reinforcing the function of DCs to elicit a stronger tumor-specific CTL response compared with the control group (85).

TNF-liked ligand 1A (TL1A) is another TNF family cytokine that acts through its receptor, death receptor 3 (DR3), to promote the differentiation of Th9 cells via an IL-2-STAT5-dependent mechanism, but not the lL-4-STAT-6 signaling axis involved in OX40-induced Th9 cell differentiation (77). More interestingly, Richard AC et al. showed that activated STAT5 was sufficient to antagonize the inhibitory effects of IL-6 in the induction of Th9 cell differentiation. Their observation was supported by Dong Wang et al., who found that TL1A intensified the severity of colitis by increasing the secretion of IL-9 and the Th9 cell differentiation induced by the upregulation of PU.1 (Figure 1) (78).

## The Role OF IL-2 Signaling in Th9 Cell Differentiation

IL-2 and TSLP are capable of phosphorylating STAT5 (86). Yang et al. reported that STAT5-deficient (Stat5a^fl/−^Mx-Cre) Th9 cell populations produced less IL-9 than their STAT5-sufficient (Stat5a^fl/+^Mx-Cre) counterparts and revealed direct binding of STAT5 to the *IL-9* locus (58). In contrast, the IL-2-STAT5 signaling pathway restrained Th17 cell differentiation (87). Therefore, it is reasonable to conclude that IL-2 phosphorylates STAT5 and impedes the development of Th17 cells but promotes the differentiation of Th9 cells. Leonard et al. also proved that IL-2–JAK3–STAT5 signaling played a critical role in the development of Th9 cells (88). They verified that IL-9 was produced at a lower level in IL-2^−/−^ mice, which could be reversed after the addition of exogenous IL-2. A subsequent CFSE-labeling experiment showed that IL-2 had little effect on Th9 cell proliferation, indicating that IL-2 did not regulate Th9 proliferation to induce the production of IL-9. To further explore the underlying mechanisms of Th9 cell differentiation in IL-2^−/−^ mice, these authors deleted the *Stat5* gene and found that the ability of IL-2 to induce IL-9 production was markedly abolished, suggesting that STAT5 was important for IL-2-induced Th9 cell differentiation. To determine whether STAT5 directly bound to *IL-9* locus, they performed a ChIP-Seq experiment and observed STAT5 ChIP-Seq peaks. Then, they cloned the IL-9 promoter and identified two gamma interferon activation site (GAS) motif regions. They found the activity of IL-9 promoter could be induced by IL-2, but completely abrogated after the mutation of GAS motifs. Interestingly, they also found that IL-9 mRNA expression was decreased in IL-21-treated cells in contrast to IL-2-treated cells. In further analyses and experiments, their group showed that B cell lymphoma 6 (BCL-6) was oppositely regulated by IL-2 and IL-21. Mechanistically, BCL6 was capable of binding to the IL-9 promoter, competing with STAT5, whereas IL-21 enhanced the expression of BCL6, thus inhibited the differentiation of Th9 cells (88). Therefore, IL-2 and IL-21 played an opposing role in the differentiation of Th9 cells. In addition to STAT5, IL-2 activated the p38 mitogen-activated protein kinase (MAPK) and phosphoinositide 3-kinase (PI3K) pathways, resulting in the activation of the nuclear factor (NF)-kB pathway and the downstream factors (89).

## THE Role of IL-1, IFN-γ, and IL-27 Signaling in Th9 Cell Differentiation

Interleukin 1β (IL-1β) is an upstream factor in the NF-κB signaling pathway and activated by MyD88 signal transduction (90). Recently, Végran and colleagues reported that IL-1β, combined with IRF4 and PU.1, drove the STAT1-dependent expression of IRF1, and then bound to the promoters of IL-9 and IL-21, increasing the secretion of the IL-9 and IL-21 cytokines from Th9 cells (91). Their work revealed the role of the IL-1β-STAT-1-IRF-1 axis in the differentiation of Th9 cells.

It has been reported that IFN-γ suppressed Th9 cell differentiation through IL-27 derived from dendritic cells, which was partially dependent on STAT-1 and T-bet (92). Besides, they found that with the addition of IL-27, Th9 cell differentiation was inhibited, while the secretion of IL-10, IL-21, and IFN-γ was increased. However, the inhibition of IL-9 production induced by IL-27 was independent of IL-10, IL-21, and IFN-γ (92).

## The Role OF IL-7 Signaling in Th9 Cell Differentiation

IL-7 is essential for the development and survival of naïve T cells both *in vitro* and *in vivo* (93, 94). More importantly, it has been widely reported that IL-7 was capable of promoting Th1 cell differentiation and enhancing the antitumor effect mainly based on cytotoxic T cells (95–97). Recently, Yi et al. found that IL-7 increased the differentiation and the antitumor activity of Th9 cells (98). In contrast to IL-1β, they found that IL-7 did not increase Th9 cell differentiation in the presence of TGF-β, IL-4, or a monoclonal antibody against IFN-γ. However, when naïve CD4^+^ T cells were pretreated with IL-7 for 48 h and then were cultured under Th9 cell differentiation condition, the expression of IL-9 and IL-21 was continuously increased compared with naïve CD4^+^ T cells without IL-7 pretreatment. They also tested IL-2, IL-15, and other γC receptor family cytokines, but no similar effect was observed, indicating that IL-7 was specific to Th9 cell differentiation. To determine the critical signaling pathways involved in IL-7-induced Th9 cell differentiation, they performed a gene microarray assay for IL-7-pretreated and unpretreated CD4^+^ T cells. The results showed that the expression of some histone acetyltransferase-encoding genes was increased in IL-7-pretreated cells. Further experiments proved that the expression of GCN5 and p300 was increased in the presence of IL-7, leading to an increment in histone 3 (H3) acetylation at the IL-9 promoter locus. These effects were almost completely reversed by the p300 inhibitor. Then, they showed that the STAT5 and PI3K-AKT-mTOR signaling pathways were critical in mediating the expression of p300 in IL-7-pretreated cells. Through bioinformatics analysis, they identified two Forkhead box transcription factor-binding sites that regulated IL9 expression. Since Foxo1 has been reported to be an important downstream factor of PI3K-AKT-mTOR signaling and competed with Forkhead box protein O1 (Foxp1) for binding to the IL-7r promoter, the authors wondered whether Foxo1 and Foxp1 might regulate IL-9 expression. To test their hypothesis, they overexpressed Foxo1 and knocked down Foxp1 and found that Th9 cell differentiation was significantly promoted. The same results were observed in *in vivo* experiments, indicating that Foxo1, which was activated by p300, might be a positive regulator of IL-7-induced Th9 cell differentiation, whereas Foxp1 served as a negative regulator.

## The Anti-Tumor Effect of Th9 Cells

In recent years, several groups have reported that Th9 cells were effective in eliciting anti-tumor immune responses and suppressing tumor growth. Up to now, the anti-tumor properties of antigen-specific Th9 cells have been studied in several different tumors, including melanoma, lung adenocarcinoma, colon cancer, and breast cancer (Table 2) (28, 29, 76, 85, 107, 109, 113–115).

**Table 2:** Functions of Th9 cells and1 IL-9 on different tumors.

**System**	**Type of cancers**	**Role of Th9 cells or IL-9**	**References**
Hematologic	B-cell non-Hodgkin's lymphoma (NHL)		(99–105)
	Chronic lymphocytic leukemia (CLL)		
	T-cell leukemia(ACTL)		
	Hodgkin lymphoma(HD)	Pro-tumor (IL-9)	
	Cutaneous T-Cell Lymphoma (CTCL)		
	Anaplastic large-cell lymphoma (ALCL)		
	NKT cell lymphoma		
Digestive system	Hepatocellular carcinoma	Pro-tumor (Th9)	(106)
	Colon cancer	Anti-tumor (Th9)	(85, 107, 108)
Dermal system	Melanoma	Anti-tumor (Th9)	(10, 27, 28, 91, 107, 109–111)
			
			
Respiration system	Lung cancer	Pro/anti-tumor (Th9)	(106)
			(76, 112)
			
Reproductive System	Breast cancer	Anti-tumor (Th9)	(113)

*NHL, B-cell non-Hodgkin's lymphoma; CLL, chronic lymphocytic leukemia; ALCL, Anaplastic large-cell lymphoma; CTCL, Cutaneous T-cell lymphoma; HD, Hodgkin lymphoma; ATCL, T-cell leukemia*.

The seminal observation was reported by Purwar et al. (28), who incidentally found that B16F10 melanoma growth was inhibited in retinoid-related orphan receptor γ (ROR^−/−^γ)-deficient mice, which presented a greater number of infiltrating CD4^+^ and CD8^+^ T cells at tumor sites and secreted a high level of IL-9 compared with their *Rorc*^+/+^ch counterparts. The neutralization of IL-9 successfully reversed this effect, suggesting an antitumor role of IL-9 against melanoma, in contrast to previous studies involving hematological cancers (99–101, 108). More importantly, differentiated Th cells, including Th1, Th2, Th9, and Th17 cells, generated from the naïve CD4^+^ T cells of OT-II mice were transferred to B16-OVA tumor-bearing mice. The results showed that tumor growth presented the greatest delay under treatment with Th9 cells compared with all the other CD4^+^ T cell subsets, including Th1 and Th17 cells. Lu and colleagues (27) also supported this result. They found that the adoptive transfer of tumor-specific Th9 cells elicited a strong antitumor response in B16-OVA-bearing mice and eradicated tumor foci in a lung tumor metastasis model. Recently, another study from Lu and Yi (10) revealed the underlying mechanism of these excellent effects. They found that Th9 cells were less exhausted than Th1 cells but highly cytolytic because of the upregulation of *Eomes* expression. Initially, Lu speculated that Th9 cells might be a kind of early memory T cells or similar to Th17 cells, possessed greater antiapoptotic capacity to maintain their persistence *in vivo*. However, gene profile analysis, GSEA and FACS analysis indicated that Th9 cells skewed away from early memory lineage and had lower expression level of genes for stemness (10). Surprisingly, they found that Th9 cells possessed a hyperproliferative feature, enabling Th9 cells to persist for a long period *in vivo*, and the persistence of Th9 cells depended on hyperactivation of NF-kB signaling mediated by Traf6 (10).

## The Mechanisms of Th9 Cell Induced Anti-Tumor Effects

**Figure 2:**
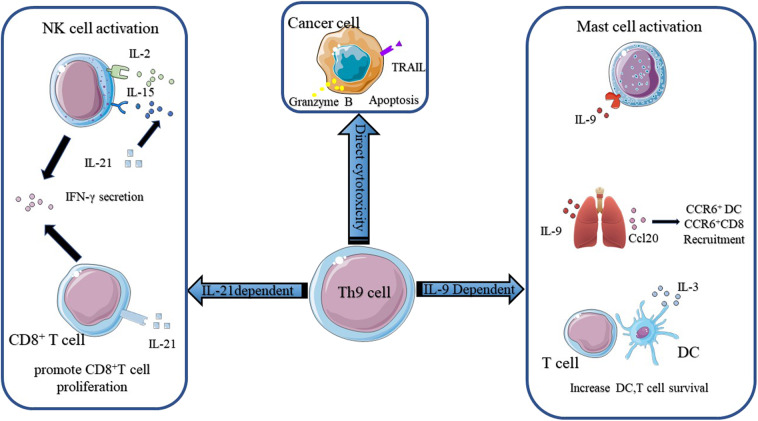
The mechanisms of Th9 cells in anti-tumor immunity. The anti-tumor function of Th9 cells mainly relies on IL-9 and IL-21. IL-9 activates mast cells and enhances their cytotoxic capacity. IL9 also activates epithelial lung cells to produce CCL20, which attracts CCR6^+^ DC and CCR6^+^ CD8^+^ T cells into the tumor bed. Besides, IL-9 increases DC and T cell survival. Th9 cells derived IL-21 promotes CD8^+^ T cell proliferation and increases NK cytolytic functions. IL-21 also induces NK and CD8^+^ T cells secretion of IFN-γ. Figures were produced using Servier Medical Art https://smart.seriver.com.

According to Puwar (28), the antitumor effect of Th9 cells mainly relied on the activation of mast cells through IL-9 but not adaptive immunity since they found that anti-IL-9 treatment inhibited tumor growth in Rag1^−/−^ C57BL/6 mice, which lacked T and B cells, whereas anti-IL-9 treatment had no influence on tumor progression in mast-cell-deficient mice injected with B16F10 melanoma cells and LLC-1 cells (Figure 2). The function of mast cells in the rejection of tumor growth was confirmed by Abdul-Wahid et al. (109). Their group constructed a vaccine that successfully elicited a Th9 cell response. In further experiments, they found that inhibiting the activity of mast cells with cromoglycate or depleting mast cells with anti-CD117 antibodies reversed the anti-tumor efficacy of the vaccine. However, the role of mast cells in the immune response is ambiguous. It has been reported that Treg cells were capable of recruiting mast cells through IL-9 and inducing an immunosuppressive milieu to protect the host (102). In addition, mast-cell infiltration was associated with a poor outcome in prostate cancers, follicular lymphoma, Hodgkin lymphoma, and Merkel cell carcinomas (116–119). These effects were attributed to the release of tumor angiogenesis factors and the recruitment of macrophages by mast cells.

The anti-tumor effect of Th9 cells also required innate and adaptive immunity. Lu and colleagues (27) found that the anti-tumor efficacy of Th9 subsets was independent of mast cells but was highly correlated with CCL20, the ligand of CCR6, derived from bronchial and alveolar epithelial cells. They demonstrated that Th9 cells transfer resulted in the recruitment of DC cells via the CCL20-CCR6 axis and increased the capacity of antigen uptake and presentation. Then DC cells migrated to tumor-draining lymph nodes (TDLNs) and primed CD8^+^ T cells in the TDLNs through the cross-presentation of tumor antigens. The role of CD8^+^ T cells in the Th9 cell-induced anti-tumor effect observed *in vivo* was reinforced by the observation that abrogating the function of CD8^+^ T cells with anti-CD8 antibodies reversed tumor rejection in a mouse model (Figure 2). This result was reconfirmed by Zhao and colleagues (120). They reported that Dectin-1-activated DCs increased the expression of OX40L and TL1A via the NF-κB signaling pathway and promoted the differentiation of Th9 cells. Further experiments showed that Dectin-1-activated DCs elicited a robust anti-tumor effect. They postulated that the anti-tumor effect of Dectne-1-activated DCs relied on Th9 cell-induced CTL responses. To test this hypothesis, they immunized B16-OVA bearing OT-II mice with Curdlan (CurDCs), the selective agonist of Dectin-1, or OVA peptide-pulsed BMDCs. Three days later, they sacrificed the mice and collected the total leukocytes from their spleens and lymph nodes to determine the level of IL-9 and the number of Th9 cells. Indeed, higher level of IL-9 and more Th9 cells were detected in CurDC-immunized mice compared with their BMDC counterparts. Additionally, they found that CurDC-immunized mice showed stronger tumor-specific CTL activity, which could be inhibited by the administration of the IL-9-neutralizing antibody. Besides, it has been reported that IL-3 derived from Th9 cells favored the survival of DCs by upregulating the expression of the anti-apoptotic protein Bcl-xL and activating the p38, ERK, and STAT5 signaling pathways (Figure 2) (110). Végran et al. also reported that the anti-tumor effects of Th9 cells relied on the involvement of CD8^+^ T cells, but more interestingly, they found that IL-1β increased the differentiation of Th9 cells and the secretion of IL-21 via IRF1 (91). The anti-tumor effect of conventional Th9 cells induced under IL-4 plus TGF-β treatment was dependent on IL-9. However, the authors reported that the anti-tumor effect of IL-1β-induced-Th9 cells relied on IL-21 since the neutralization of IL-9 had a minor impact on anti-tumor properties under this circumstance. IL-21 is a stimulator of IFN-γ production produced by activated CD4^+^ T cells. IL-21 reinforces the ability of IL-2 and IL-15 to activate NK cells, inducing their cytolytic and secretion functions (Figure 2). In addition, IL-21 contributes to the proliferation of murine CD8^+^ T cells and the expansion of antigen-stimulated human CD8^+^ T cells via IL-15. Thus, these two studies highlighted the importance of adaptive immunity and innate immunity to the anti-tumor effect of Th9 cells.

What's more, Xue and colleagues reported that IL-1β combined with IL-4, in the absence of TGF-β, induced a non-canonical Th9 subset that was less exhausted and showed superior anti-tumor effects to classic Th9 cells induced by TGF-β and IL-4. Gene array analysis revealed the downregulation of exhaustion/inhibition markers in Th9^*IL*−4+*IL*−1β^ cells compared with classic Th9^*IL*−4+*TGF*−β^ cells, including *Ctla4, Pdcd1, Lag3*, and *NT5e*. In particular, Th9^*IL*−4+*IL*−1β^ cells presented higher expression of *Eomes* and *Tbx21* and increased expression of a *Grz* panel and *Prf1* (111), suggesting that Th9^*IL*−4+*IL*−1β^ may act as cytolytic effector T cells. The *in vitro* experiments verified that Th9^*IL*−4+*IL*−1β^ cells exerted stronger tumor-specific cytotoxicity than classic Th9^*IL*−4+*TGF*−β^ cells. Interestingly, in contrast to Végran's observations (91), the anticancer efficacy of Th9^*IL*−4+*IL*−1β^ was found to be dependent on IL-9, at least in part, since the authors did not mention the function of IL-21 in their *in vivo* experiments. The intrinsic cytolytic function of Th9 subsets was supported by Lu et al. (10). They made similar observations in their study. The global transcriptional profile showed that Th9 cells increased the gene expression of *Id*2 and *Eomes*, as previously mentioned, suggesting effector cell development. They also observed increased gene expression of a granzyme panel (Gzmb, Gzmd, Gzme, Gzmg, and Gzmn) (10). Then, they performed *in vitro* experiments to test the cytolytic functions of different Th subsets. The results showed that Th9 cells possessed the highest tumor-specific killing activity among the Th cells. They also showed that Th9-mediated-specific killing was mainly dependent on granzyme B activity, in concert with the observations made by Puwar et al. (28) (Figure 2). Furthermore, Th9 cells were observed to be hyperproliferative due to the activation of the NF-κB signaling pathway and persisted for a long time *in vivo*. In addition, two groups reported the cytotoxic properties of Th9 cells in squamous and human melanoma cell lines (121, 122). In conclusion, these studies indicated that Th9 cells could act as effector T cells and directly kill tumor cells through the secretion of granzyme and IL-9.

## The Pro-Tumor Effects of Th9 Cells

IL-9 is a crucial cytokine involved in regulating the function of Treg cells and mast cells. Treg cells and mast cells are involved in the inhibition of the immune response and the promotion of tumor development. Feng et al. reported that IL-9 correlated with Foxp3^+^ regulatory T cell- and CD117^+^ mast cell-mediated immunosuppression in B-cell non-Hodgkin's lymphoma (NHL) (Table 2) (101). They found that the number of Treg cells and mast cells as well as the expression of IL-9, were increased in B-cell NHL patients. Further experiments showed that the neutralization of IL-9 caused the downregulation of Tregs and mast cells, resulting in the inhibition of tumor growth. High expression of IL-9 was detected in chronic lymphocytic leukemia (CLL), adult T-cell leukemia (ACTL), Hodgkin lymphoma (HD), anaplastic large-cell lymphoma (ALCL) and NKT cell lymphoma by different groups (Table 2) (99, 100, 103, 108, 123). These findings suggested that IL-9 might be a potential target for the development of novel therapy strategies against hematological malignancies. However, a clinical study demonstrated that Th9 cells protected the survival of malignant T cells in Cutaneous T-Cell Lymphoma (CTCL) patients (104). Kumar et al. reported that higher accumulation of Th9 cells was detected in early and advantage CTCL patients, while the frequency of Th9 cells decreased after standard photo/chemotherapy treatment. They also found the expression of IL-9 receptor of T cells was upregulated in CTCL patients compared with that of healthy donors. Mechanistically, IL-9 reduced oxidative stress, lactic acidosis, and apoptosis of T cells and promoted the survival of malignant T cells (104). Up to now, no direct evidence has been observed to prove that IL-9 in hematological malignancies was derived from Th9 cells and the underlying mechanisms of IL-9 in various hematological malignancies development need to be further explored.

In contrast to hematological malignancies, the protumor effect of Th9 cells was reported in hepatocellular carcinoma (HCC) by Tan et al. (105) (Table 2). They found that the infiltration of Th9 cells was increased at peritumor and tumor sites compared with normal liver tissue and indicated a decreased survival period. They further explored the underlying mechanism of this effect and found that Th9 cells increased the expression of CCL20 via enhancing the phosphorylation of STAT3, which has been reported to be associated with the poor prognosis of HCC patients (105). CCL20 is a chemokine ligand which is known to promote tumor cell proliferation and migration (106). However, as suggested by Lu, tumor-infiltrating Th9 cells induced lung epithelial cells to express CCL20 and then recruited DCs to tumor sites (27). Others reported that CCL20 was capable of inducing epithelial-mesenchymal-transformation (EMT) in HCC cells, promoting the engraftment of tumor cells (124). Thus, the role of CCL20 in tumor development may be tumor specific.

Recently, Salazar et al. reported that micro-environmental Th9 cells promoted tumor metastasis in lung cancer (125). First of all, they found that accumulation of Th9 cells in human lung cancer tissue was correlated with poor survival. Then Th9 cells were co-cultured with LLC1 and induced EMT in cancer cells. In addition, their RNA sequencing data indicated the upregulation of genes related to EMT and metastasis, such as *MMP3, MMP13, PlexinA4*. These results were reproduced by IL-9 *in vitro*, indicating the pivotal role of IL-9 in promoting tumor growth and metastasis in lung cancer. Co-injection of LLC1 cells and Th9 cells promoted lung cancer growth and metastasis in Rag1^−/−^ mice, whereas neutralization of IL-9 reversed these effects (125). In addition, Shi et al. found that Th9 cells were accumulated in malignant pleural effusion (MPE) and indicated a shorter survival period, suggesting that Th9 cells might promote the development of tumors (112). *In vitro* experiments showed that IL-9 and MPE supernatants increased the proliferation of lung cancer cells, while the addition of anti-IL-9 abrogated this effect. IL-9 also inhibited the apoptosis and promoted the migration and adhesion abilities of lung cancer cells. Although Th9 cells are capable of eliminating tumor cells in different ways, it is still very likely that Th9 cells may support lung cancer progression.

## Conclusions and Prospects

The successful application of checkpoint inhibitors, especially anti-PD-1/PD-L1, underscores the potential of utilizing endogenous antitumor immunity to fight cancers. Previous studies have demonstrated that Th9 cells exhibited superior antitumor effects to the Th1 and Th17 subsets (10, 28), and the antitumor properties of Th9 cells relied on innate immunity, adaptive immunity, and the intrinsic killing capacity of Th9 cells. According to previous research, IL-9 activated mast cells and increased DC survival (28, 110). In addition, IL-9 boosted the production of CCL20 in lung tissue, leading to the recruitment of CCR6^+^ DCs and CCR6^+^ CD8^+^ T cells to the tumor bed (27). Th9 cell-derived IL-21 promoted CD8^+^ T cell proliferation and improved the cytolytic ability of NK cells. Additionally, IL-21 increased the IFN-γ secretion of both NK cells and CD8^+^ T cells (91). Qing Yi and colleagues demonstrated that Th9 cells had direct cytotoxicity to tumor cells via the secretion of Granzyme B (10). Their work also answered why Th9 cells persisted for a long time *in vivo*, even though this had been observed several years previously.

The findings regarding Th9 cells have shed light on immunotherapy for cancers, here we list several works that need to be done in the future. First, it is necessary to explore whether Th9 cells also present strong anti-tumor properties against other solid tumors. In addition, as Th9 cells are capable of promoting DC survival and recruiting DCs to the tumor bed, DC cancer vaccines could be a promising candidate, combined with Th9 cells, to stimulate anti-tumor immunity in tumor patients. In addition to their anti-tumor properties, Th9 cells have been found to promote tumor development, especially hepatocellular carcinoma and lung cancer (106, 112, 125). To better manipulate Th9 cells for the treatment of cancers, it is essential to explore why Th9 cells play different roles in different cancers and the underlying mechanisms in various conditions. Last but not least, Th9 cells and IL-9 are pro-inflammatory factors and are closely related to autoimmune diseases such as SLE, MS, IBD, RA and psoriasis. Consequently, the immune-related toxicity to the host must also be taken into consideration when the Th9 cells are transferred to the patient.

Since the discovery of Th9 cells in 2008, an extensive array of signaling molecules and transcription factors involved in Th9 cell differentiation have been revealed. However, it is difficult to obtain a comprehensive understanding of the transcriptional regulation of the Th9 subset due to the ambiguity and overlap of crucial cytokines required for Th9 transcriptional regulation with other T helper subsets. To manipulate Th9 cells therapeutically, much more effort will be required to obtain a better understanding of Th9 cell development and function.

## Author Contributions

TC, JG, and ZhenC wrote the manuscript. BL, YS, SW, ZhanW, and LS provided expertise and advice. ZeW, HZ, and YW critically read the manuscript. ZY and ZhiC supervised the project.

### Conflict of Interest

The authors declare that the research was conducted in the absence of any commercial or financial relationships that could be construed as a potential conflict of interest.
